# An Open-Label Pilot Study to Assess the Efficacy and Safety of Virgin Coconut Oil in Reducing Visceral Adiposity

**DOI:** 10.5402/2011/949686

**Published:** 2011-03-15

**Authors:** Kai Ming Liau, Yeong Yeh Lee, Chee Keong Chen, Aida Hanum G. Rasool

**Affiliations:** ^1^Healthy Lifestyle Cluster, Advanced Medical and Dental Institute, Universiti Sains Malaysia, Pulau Pinang 11800, Malaysia; ^2^Department of Medicine, School of Medical Sciences, Universiti Sains Malaysia, Kelantan 16150, Malaysia; ^3^Department of Sport Sciences, School of Medical Sciences, Universiti Sains Malaysia, Kelantan 16150, Malaysia; ^4^Department of Pharmacology, School of Medical Sciences, Universiti Sains Malaysia, Kelantan 16150, Malaysia

## Abstract

*Introduction*. This is an open-label pilot study on four weeks of virgin coconut oil (VCO) to investigate its efficacy in weight reduction and its safety of use in 20 obese but healthy Malay volunteers. *Methodology*. Efficacy was assessed by measuring weight and associated anthropometric parameters and lipid profile one week before and one week after VCO intake. Safety was assessed by comparing organ function tests one week before and one week after intake of VCO. Paired *t*-test was used to analyse any differences in all the measurable variables. *Results*. Only waist circumference (WC) was significantly reduced with a mean reduction of 2.86 cm or 0.97% from initial measurement (*P* = .02). WC reduction was only seen in males (*P* < .05). There was no change in the lipid profile. There was a small reduction in creatinine and alanine transferase levels. *Conclusion*. VCO is efficacious for WC reduction especially in males and it is safe for use in humans.

## 1. Introduction

Developing countries including many Asian countries catch up rapidly in the prevalence of obesity with higher health risks at a lower body mass index (BMI). The latest report from Malaysian Non-Communicable Diseases (NCD) surveillance in 2007 demonstrated that among the adult males, 30.9% were overweight and 13.9% were obese, whereas among adult females, 32.4% were overweight and 18.8% were obese (the results were based on BMI ≥ 25 kg/m^2^ for overweight and BMI ≥ 30 kg/m^2^ for obese) [1]. The modernization of society with reduced levels of physical activity and increased dietary intake of carbohydrate and highly saturated fat accounted for the rapid growth in obesity epidemic. The fat accumulating in the abdomen, known as visceral adiposity was associated with increased cardiovascular risk, insulin resistance, and dyslipidemia [2, 3]. Waist circumference (WC), BMI, and waist-hip ratio (WHR) are useful surrogate anthropometric markers for general and visceral adiposity [4–6].

 Coconut (*Cocos Nucifera*) is commonly used in Malaysia and other neighbouring countries including Thailand and Philippines as a food source and its oils are used as complementary medicine. The oil extracted from fresh coconut meat (virgin coconut oil) contains more medium chain fatty acids (MCFAs) (70–85%) (Table 1) compared to other coconut oils [7]. MCFAs are easily oxidized lipids and are not stored in adipose tissue unlike long chain fatty acids (LCFAs). Thus, coconut oil containing mainly MCFAs with little or no LCFAs may provide an ideal food source for weight reduction [8, 9]. Furthermore, epidemiologic studies coming from the African and South Pacific populations whose diets contain coconuts have revealed no association between coconut oil ingestion and obesity or dyslipidemia [10, 11]. Therefore, we conducted this open label pilot study to determine the efficacy of VCO on weight reduction, anthropometric parameters, and lipid profile in obese healthy volunteers and to assess for its safety by evaluating changes in biochemistry and organ functions.

## 2. Materials and Methods

### 2.1. Subjects

Free living volunteers above the age of 20 years within the university compound (Universiti Sains Malaysia or USM) were screened for their weight, height, body mass index (BMI), and waist circumference (WC). Overweight subjects were defined as BMI ≥ 23 kg/m^2^, and obese subjects were defined as BMI ≥ 25 kg/m^2^. Abdominal obesity was present when the waist circumference was ≥90 cm for men and ≥80 cm for women. The criteria for overweight, obese, and abdominal obesity were adapted from the Asia-Pacific report on obesity in 2000 [12]. Subjects were included in this study if they were between the ages of 20 to 60 years old and have BMI more than 23 kg/m^2^. Exclusion criteria include presence of any medical or surgical illnesses, pregnant women, those who drink any alcohol containing beverages in any amount and previous history of intolerance to coconut oil. The study was approved by the Human Ethics Committee of USM. 

 We have screened 65 subjects over a period of 6 months in 2008. Finally 20 subjects who agreed and satisfied all inclusion and exclusion criteria participated in the study after informed consent. The following parameters were evaluated at “week one of study” defined as one week prior to VCO intake and again on “week six of study” defined as one week after completion of 4 weeks of VCO intake.

### 2.2. Anthropometric Evaluation

The body composition of each subject including body weight, body fat percentage, fat mass (FM), and fat-free mass (FFM) was measured with subjects in light clothing using Tanita body composition analyzer (Model TBF-410, Tanita Corporation). This analyzer has a maximum capacity of 200 kg and a precision of 0.1 kg. All the subjects were instructed to fast overnight and to consume 250 mL of plain water half an hour before the actual measurement to ensure adequate hydration. 

 Heights were measured with the subjects in bare-foot using an extendable measuring rod (Seca, Model 220/221) with a maximum measuring length of 200-cm and a precision of 0.1 cm. WC was measured at the mid point between the last rib and the anterior superior iliac spine with subjects standing upright using the same nonextendable measuring tape. Hip circumference (cm) was measured at the level of greater trochanter of the femur. WHR was calculated by dividing the waist circumference (cm) by the hip circumference (cm) and the result presented as a ratio. The BMI was calculated by dividing the body mass (kg) by the square of height (m).

### 2.3. Dietary Evaluation

Before the study was commenced, a 24-hour dietary recall was applied to all subjects. Food frequency questionnaire and images depicting different quantities of food were used to assist participants in assessing the amount of food consumed. The participants were instructed to continue with the same pattern of diet intake for the next 5 weeks. Participants were also advised to drink adequate amount of water throughout the study period.

### 2.4. Physical Activity Evaluation

Each participant was required to report their usual physical activities (past 1 month) before the commencement of the study. The participants were instructed to continue with the same amount of physical activity each day for the next five weeks.

### 2.5. Lipid Profiles

In each subject, venous blood was taken in the morning following a 12-hour overnight fast. Triglyceride (TG), total cholesterol (TC), high-density lipoprotein (HDL), and low-density lipoprotein (LDL) were determined in triplicate for each sample using a blood chemistry analyzer (Model WS-ROCHE912).

### 2.6. Safety Evaluation

Volunteers were requested for additional venous blood samples to assess for electrolytes, glucose level, renal function, and liver function. Tests for electrolytes included sodium, potassium, calcium, phosphate, and uric acid levels. Tests for renal function included urea and creatinine levels. Tests for liver function included levels for albumin, total bilirubin, aspartate transferase (AST), alanine transferase (ALT), and alkaline phosphatase (ALP). 

 Of all the 20 participating subjects, only 16 subjects had complete blood results (including electrolytes, glucose level, renal function, and liver function tests) to assess for safety after usage of VCO. Two subjects refused further blood taking on week six of study, and the other two subjects had incomplete blood results on week six of study, and were thus excluded from analysis.

### 2.7. Virgin Coconut Oil

The subjects were prescribed with VCO 1 week after initial evaluation and continued over the next four weeks. The VCO was sourced from a local agricultural product company and was produced using freeze-thawed method with no preservatives or additives added. The oil was certified suitable for consumption by the Malaysian Agricultural Research and Development Institute (MS ISO 9001:2000 and MS ISO/IEC 17025 certified). The composition profile of VCO was determined by an independent and accredited laboratory (Food Quality Research Unit, Universiti Kebangsaan Malaysia) and is presented in Table 1. 

 The prescribed dosage was 30 mL per day taken in three divided doses, half an hour before each meal. Calculated based on the amount of lauric acid found in human mother's milk, the suggested daily intake of 24 g of lauric acid in an average adult is equivalent to 30 mL per day of VCO [13]. In addition, our early experience with earlier volunteers suggested that 30 mL per day was a more tolerable dose compared to higher doses without any side effects.

### 2.8. Data and Statistical Analysis

Mean and standard deviations were calculated for all numerical measurements taken at week one and week six of study. Paired *t*-test analysis was then used to test for any differences in all measured variables at week one and week six of study with the significance level set at 95% confidence interval (CI) and *P* < .05. To assess for possible differences between gender, measured variables at week one and week six of study for females and males were analysed separately and their mean differences were calculated and compared. All statistical analyses were performed using SPSS version 18.0 (SPSS Inc, IL, Chicago).

## 3. Results

The volunteers were relatively young with a mean age of 40.5 ± 8.87 years (age range between 24 to 51 years). All subjects enrolled were of Malay ethnic origin. 20 subjects completed the study, out of which 13 were females and 7 were males. All enrolled volunteers were obese by definition (BMI ≥ 25 kg/m^2^) with 13 subjects having BMI above 30 kg/m^2^. Of these 13 subjects, 7 were males. All enrolled males had waist circumference above 90 cm and all enrolled females had waist circumference above 80 cm except for one subject with a waist circumference of 77 cm. 

 The results for all measured variables to assess for efficacy at week one and week six of study are presented in Table 2. Paired *t*-test analysis showed that only waist circumference was significantly reduced after one month of VCO with a mean reduction of 2.87 ± 4.95 cm or 0.97% from initial measurement (*P* = .02). All other variables showed a nonsignificant reduction in their mean values except for FFM and HDL which showed a nonsignificant increase. The waist circumference reduction was only significantly seen in males with a mean reduction of 2.61 ± 2.17 cm (*P* = .02; 95% CI 0.61–4.62) but not in females (*P* = .10) even though the reduction was larger (mean 3.00 ± 6.03 cm) in females. A subgroup analysis comparing seven males and seven females with BMI > 30 showed that the WC reduction was seen with males but not females (mean 2.61 cm versus 1.14 cm; *P* = .019). When comparing seven females with BMI ≥ 30 versus six females with BMI < 30, more reduction in WC was seen in the subgroup BMI < 30 (mean reduction 4.80 ± 6.10 cm versus 1.14 ± 6.20 cm) but the reduction was not statistically significant (*P* = .153). The weight reduction (mean 0.54 ± 1.38 Kg) and BMI reduction (mean 0.24 ± 0.55 kg/m^2^) were nonsignificantly higher in males when compared to females. In addition, there were nonsignificant increases in total cholesterol (mean 0.08 ± 0.61 mmol/L), LDL (mean 0.12 ± 0.37 mmol/L), and HDL (mean 0.13 ± 0.37 mmol/L) which were not seen in the females. FFM, however, showed a nonsignificant increase in females (mean 0.93 ± 3.24 kg) but not in the males. 

 The results for all measured variables to assess for safety after VCO consumption are presented in Table 4. The electrolytes and glucose levels did not have significant changes before and after VCO consumption. The creatinine but not urea level showed a significant reduction after VCO consumption (mean 6.00 ± 5.19 mmol/L;  *P* < .001). Liver function tests did not have significant changes except for ALT level (mean 3.06 ± 4.40 mmol/L; *P* = .01) which reduced after VCO consumption.

## 4. Discussion

Coconut oil belongs to a group of vegetable oils that has an abundance of lauric acid. A study has shown that consumption of solid fat rich in lauric acid resulted in a more favourable serum lipid profile in healthy men and women than with solid fat containing *trans-*fatty acids [14]. An emerging medicinal product of importance from coconut is virgin coconut oil (VCO) which is cheap, easily available, and widely used as over-the-counter complementary medicine in the tropics and many foreign markets [15, 16]. Of all different types of coconut oils, VCO contains the highest proportion of medium chain fatty acids, with MCFA content being as high as 85.1% in VCO (Table 1). Hence this oil naturally contains a mixture of MCFA and LCFA in a ratio of 3 : 1. MCFAs are rapidly absorbed in the intestines even without catalyzation by the pancreatic lipase enzyme. LCFAs, on the other hand, required pancreatic lipase for absorption. They are carried by the lymph to the systemic circulation in chylomicrons and eventually reach the liver where they either undergo beta oxidation, biosynthesis to cholesterol, or are repackaged as triglycerides. MCFAs are carried by the portal vein to the liver where they are rapidly oxidized to energy. Unlike LCFAs, MCFAs do not enter the cholesterol cycle and they are not deposited in fat depots [7]. 

 This open label pilot study attempted to find out the efficacy of VCO on reduction of weight and anthropometric markers of obesity in participants after 4 weeks of 30 mL in three divided doses daily VCO consumption. All the participants in this study were instructed to continue their normal daily diet and physical activities to minimize possible weight reduction and change in blood lipid profile which could be attributed to reduced calorie intake or increased energy consumption. Only WC was significantly reduced after four weeks of VCO consumption with a mean reduction of 2.87 ± 4.95 cm or 0.97% reduction from baseline measurement. There was a nonsignificant decrease in FM and body fat percentage with a nonsignificant increase in FFM. This indicated that VCO consumption reduced body fat especially abdominal fat since WC was significantly decreased. The effects on triglyceride, total cholesterol, LDL, and HDL were almost negligible indicating that VCO did not affect lipid profiles despite being an oil-based food source. 

 When the differences were analyzed according to gender, WC was significantly reduced in males but not in females. This difference was still seen only in men when analyzed in subgroups of males and females with BMI ≥ 30. The significant reduction in WC may be attributed to the nonsignificant reduction in weight and BMI among the males. This finding was important since for a given WC, the visceral adiposity was higher in males of Asian ethnic [5, 17]. The significant reduction of WC is considered modest given the short duration of this study. Furthermore, all males in the cohort are larger (BMI ≥ 30 kg/m^2^) and therefore are more resistant to weight loss. Few studies exist in males on the optimal weight or BMI or WC reduction for a given weight loss intervention. The “Gutbuster” programme in Australia uses waist circumference as a target to encourage weight management in men, with a target of 1% waist reduction a week [18]. Colman et al. found that a loss of 9 kg weight reduced waist circumference by 7 cm in men [19]. A nonsignificant increase in total cholesterol, LDL, and HDL were also observed but the overall increase was too small. The effects of VCO on lipid profiles may need a longer time to be observed. An increase in HDL level but a reduction in total cholesterol and LDL levels after consumption of coconut oil was reported in experimental animals [20]. Furthermore, the increase in LDL may be due to a different form of lipoprotein not associated with increase in cardiovascular risk since there were animal studies which demonstrated that the increase in LDL level after VCO was not associated with aortic atherosclerosis [21]. 

 In contrast, females exhibited different anthropometric profiles and lipid profiles after VCO consumption when compared to their male counterparts. Even though the reduction in WC was larger compared to males it was not statistically significant. When comparing females with BMI ≥ 30 and BMI < 30, the reduction in WC was greater in females with BMI < 30 but it was not statistically significant. The larger reduction in WC was not associated with a decrease in BMI or weight but was associated with a nonsignificant increase in FFM and a nonsignificant decrease in FM and body fat percentage. The data appeared to indicate that females in general lose more of their body fat with VCO and females with a lower BMI may lose more abdominal fat. This was not reflected on their BMI or WHR in contrast with their male counterparts. This supports the evidence that different indices are applicable to different gender and ethnic groups [6, 22]. However, the insignificant reduction in WC can also be explained by the relatively high-standard deviation suggesting that there was a high variability of WC reduction among females who took VCO (Table 3). In addition, the total cholesterol and LDL appeared to decrease in females who consume VCO with triglycerides and HDL almost unchanged. A study using coconut oil in obese women demonstrated a reduction of abdominal fat with unchanged lipid profiles providing support for similar findings in the current study [9]. This also reflected that females benefited from VCO in a manner different from males [23, 24]. 

 This pilot study also attempted to assess the safety aspects of using VCO especially biochemical changes and organ functions including the renal and liver functions. Results have shown that all measured variables did not demonstrate any increase from baseline but interestingly two biochemical markers were shown to reduce after being given VCO. These markers were creatinine and ALT levels. Animal studies did not have any similar findings as in humans but this may have been because of the differences in the type of coconut oil and doses used [25]. This finding in the humans however cannot be explained and merits further study. 

 There were some limitations to this study. Firstly, there was no long-term followup on the weight, anthropometric, and lipid profile in the subjects. The full effects of VCO may not be realized without a longer duration of followup. Even though no serious side effects were reported from the volunteers after one month of VCO consumption, a long-term followup will be able to determine the safety of using VCO for long periods. Secondly, the duration of VCO consumption was probably too short as this is a pilot study. A longer period of VCO consumption may reveal more clinical differences not shown in a short-term study. In addition, a longer period of study can assess the tolerability of subjects toward coconut oil. It appeared that one month of VCO was well tolerated by all the subjects in this study. Thirdly, the number of subjects was too small contributing to the many nonsignificance results seen. Finally, the open-label design and lack of control group may introduce bias to the results. Therefore, a properly designed randomized placebo-controlled trial should be performed to further confirm the beneficial effects of VCO. 

 In conclusion, VCO is a cheap oil source containing high concentration of MCFAs which in the current study had shown beneficial effect in WC reduction especially in males without any deleterious effect to the lipid profile. VCO is also safe to use for the period of study without any deleterious effects on biochemical and organ functions.

##  Conflict of Interests 

All authors did not report any conflict of interests.

## Figures and Tables

**Table 1:** Fatty acid profiles of virgin coconut oil.

	Fatty acid profile	Concentration (%)
C6	Caproic	2.215
C8	Caprylic	12.984
C10	Capric	6.806
C11	Undecanoic	0.028
C12	Lauric	47.280
C13	Tridecanoic	0.030
C14	Myristic	15.803
C15	Pentadecanoic	0.006
C16	Palmitic	6.688
C16 : 1	Heptadecanoic	0.011
C17	Stearic	0.011
C18	Oleic	1.481
C18 : 1n9c	Elaidic	5.073
C18 : 1n9t	Linoleic	0.231
C18 : 2n6c	Linolelaidic	1.168
C18 : 2n6t	*γ*-Linolenic	0.045
C18 : 3n6g	*α*-Linolenic	0.007
C18 : 3n3a	Arachidic	0.013
C20	Cis-11-Eicosenoic	0.039
C20 : 1n9	Behenic	0.039
C22	Cis-13,16-Docisadienoic	0.006
C24	Lignoceric	0.020

Source: Food Quality Research Unit, Universiti Kebangsaan Malaysia (UKM), Kuala Lumpur, Malaysia.

**Table 2:** Comparison of anthropometric measurements and lipid profile values for the 20 obese volunteers at week one and week six of study.

Parameters	Week one mean (SD)	Week six mean (SD)	Mean difference (SD)	95% Confidence interval	*P* value
Weight (kg)	82.77 (21.44)	82.53 (21.78)	0.23 (1.08)	−0.27, 0.73	.35
Body mass index (kg/m^2^)	32.51 (5.65)	32.40 (5.69)	0.10 (0.68)	−0.22, 0.42	.51
Waist circumference (cm)	102.64 (12.56)	99.78 (12.56)	2.87 (4.95)	0.55, 5.18	.02*
Waist-Hip Ratio	0.92 (0.05)	0.91 (0.07)	0.02 (0.05)	−0.01, 0.04	.13
Body fat percentage (%)	39.91 (7.16)	39.31 (6.51)	0.60 (2.87)	−0.74, 1.94	.36
Fat mass (kg)	33.33 (12.64)	32.78 (12.01)	0.54 (2.72)	−0.73, 1.82	.38
Fat-free mass (kg)	49.20 (12.26)	49.77 (12.28)	−0.58 (2.75)	−1.86, 0.71	.36
Triglyceride (mmol/L)	1.36 (0.61)	1.22 (0.41)	0.14 (0.56)	−0.12, 0.40	.28
Total Cholesterol (mmol/L)	5.46 (0.85)	5.35 (0.68)	0.11 (0.74)	−0.24, 0.45	.53
LDL (mmol/L)	3.33 (0.88)	3.25 (0.83)	0.08 (0.91)	−0.35, 0.51	.70
HDL (mmol/L)	1.52 (0.41)	1.55 (0.41)	−0.03 (0.51)	−0.27, 0.21	.79

*P value is significant when *P* < .05, SD = standard deviation.

**Table 3:** Comparison of mean differences in anthropometric measurements and lipid profile values in 7 males and 13 females after VCO consumption.

Parameters	Male (*n* = 7)			Female (*n* = 13)		
	Mean difference (SD)	95% Confidence interval	*P* value	Mean difference (SD)	95% Confidence interval	*P* value
Weight (kg)	0.54 (1.38)	−0.73, 1.82	.34	0.06 (0.89)	−0.48, 0.60	.81
Body mass index (kg/m^2^)	0.24 (0.55)	−0.27, 0.74	.29	0.03 (0.75)	−0.42, 0.48	.89
Waist circumference (cm)	2.61 (2.17)	0.61, 4.62	.02*	3.00 (6.03)	−0.64, 6.64	.10
Waist-Hip Ratio	0.02 (0.03)	−0.01, 0.05	.14	0.02 (0.06)	−0.02, 0.05	.32
Body fat percentage (%)	0.17 (1.50)	−1.22, 1.56	.77	0.83 (3.42)	−1.24, 2.90	.40
Fat mass (kg)	0.07 (1.47)	−1.28, 1.43	.90	0.80 (3.23)	−1.15, 2.75	.39
Fat-free mass (kg)	0.09 (1.49)	−1.29, 1.46	.88	−0.93 (3.24)	−2.89, 1.02	.32
Triglyceride (mmol/L)	0.38 (0.79)	−0.35, 1.11	.25	0.01 (0.36)	−0.21, 0.23	.91
Total Cholesterol (mmol/L)	−0.08 (0.61)	−0.65, 0.49	.74	0.21 (0.81)	−0.28, 0.69	.37
LDL (mmol/L)	−0.12 (0.37)	−0.47, 0.22	.27	0.19 (1.10)	−0.47, 0.85	.55
HDL (mmol/L)	−0.13 (0.37)	−0.39, 0.13	.41	0.02 (0.60)	−0.34, 0.38	.90

*P value is significant when *P* < .05, SD = standard deviation.

**Table 4:** Levels for electrolytes, glucose, renal function tests, and liver function tests to assess for safety in 16 volunteers at week one and week six of study.

Parameters	Week one mean (SD)	Week six mean (SD)	Mean difference (SD)	95% Confidence interval	*P* value
Sodium (mmol/L)	138.75	140.25	−1.50 (4.62)	−3.96, 0.96	.21
Potassium (mmol/L)	4.19	4.32	−0.12 (0.40)	−0.34, 0.09	.23
Calcium (mmol/L)	2.34	2.31	0.03 (0.17)	−0.06, 0.12	.53
Phosphate (mmol/L)	1.24	1.18	0.06 (0.21)	−0.05, 0.17	.27
Uric acid (mmol/L)	276.56	257.12	19.44 (37.75)	−0.68, 39.55	.06
Glucose (mmol/L)	4.58	4.40	0.18 (0.56)	−0.12, 0.48	.21
Urea (mmol/L)	4.39	4.38	0.01 (1.08)	−0.56, 0.59	.96
Creatinine (*μ*mol/L)	88.62	82.62	6.00 (5.19)	3.23, 8.76	<.001
Albumin (g/dL)	44.62	44.50	0.12 (2.09)	−0.99, 1.24	.80
Total bilirubin (*μ*mol/L)	7.87	8.25	−0.37 (3.50)	−2.24, 1.49	.67
AST (U/L)	21.06	19.31	1.75 (3.59)	−0.16, 3.66	.07
ALT (U/L)	26.56	23.50	3.06 (4.40)	0.72, 5.41	.01
ALP (U/L)	70.19	71.19	−1.00 (6.33)	−4.37, 2.37	.54

*P value is significant when *P* < .05, SD = standard deviation.
